# Rapidly-Dissolving Silver-Containing Bioactive Glasses for Cariostatic Applications

**DOI:** 10.3390/jfb9020028

**Published:** 2018-04-11

**Authors:** Omar Rodriguez, Adel Alhalawani, Saad Arshad, Mark R. Towler

**Affiliations:** 1 Department of Mechanical & Industrial Engineering, Ryerson University, Toronto, ON M5B 2K3, Canada; omaralejandro.rodrig@ryerson.ca (O.R.); adel.alhalawani@ryerson.ca (A.A.); saad.arshad@ryerson.ca (S.A.); 2 Li Ka Shing Knowledge Institute, St. Michael’s Hospital, Toronto, ON M5B 1W8, Canada

**Keywords:** bioactive glass, dental caries, remineralization, microhardness, cavity bacteria

## Abstract

A novel bioactive glass series containing incremental amounts of silver oxide was synthesized, ground down, and subsequently incorporated into a dentifrice for the purpose of reducing the incidence of dental caries and lesion formation. Three glasses were synthesized using the melt quench route: Si-Control (70SiO_2_–12CaO–3P_2_O_5_–15Na_2_O, mol %), Si-02 and Si-05, where 0.2 and 0.5 mol % Ag_2_O were substituted, respectively, for SiO_2_ in Si-Control. The glasses were then ground, sieved, characterized, and dissolved in Tris buffer solution (pH = 7.30) for 6, 12, and 24 h, with the pH of the resultant solution being recorded and the ions that were released into solution quantified. Samples of each glass were subsequently embedded into a non-fluoridated, commercially available toothpaste which was then used to brush resin-mounted lamb molars which, up to the point of testing, had been stored in a 1.0 M HCl solution. Knoop microhardness measurements of the molars were recorded before and after brushing to determine the presence of remineralization on the surface of the teeth (surface hardness loss of 37%, 35%, and 34% for Si-Control, Si-02 and Si-05, respectively, after 24 h). Four oral cavity bacterial strains were isolated through swabs of the inner cheek, gums, and teeth surfaces of three volunteers, and placed on agar discs. Of each glass, 0.5 g was placed onto the discs, and the resultant inhibition zones were measured after 6, 12, and 24 h. Si-05 performed better than Si-02 on two strains after 24 h, while exhibiting similar behavior for the remaining two strains after 24 h; the largest inhibition zone measured was 2.8 mm, for Si-05 after 12 h. Si-Control exhibited no antibacterial effect at any time point, providing evidence for the role of silver oxide as the antibacterial component of these glasses.

## 1. Introduction

Dental caries, known as tooth decay, is among the most prevalent diseases worldwide [1]. It is caused by internal factors, such as saliva secretion and components, nutritional and hormonal status, microbial flora colonizing the teeth, and tooth surface morphology and external factors, such as oral hygiene, fluoride availability, and diet [2]. Dental caries causes pain, and may result in serious infection, hospitalization, and fatality under extreme circumstances [3]. Bacteria, such as *Streptococcus mutans,* exist within plaque buildup and process fermentable carbohydrates to produce weak acids [4]. These acids cause calcium, carbonate, and phosphate ions to leach out of the enamel and dentine phases of the tooth, weakening the mineral and resulting in decay [4,5]. This process can be reversed by buffering the oral environment and restoring it to its original pH; saliva being an effective natural buffer [1]. Demineralization and remineralization occur in a continuous cycle; if the pH is not restored or if the ions needed for remineralization are not available, demineralization dominates, resulting in lesions, and subsequently, cavities [6].

Dentifrices are used to impede the erosion of the tooth surface [7]. A dentifrice is a preparation that is generally used with a toothbrush in order to clean or polish teeth [8]. Modern dentifrices often claim various beneficial effects, including plaque removal, anti-caries, and anti-bacterial effects and remineralization [8]; the latter promoted by fluoride compounds, as well as strontium and potassium compounds [7,9]. Stannous fluoride (SnF_2_), an ingredient in many toothpastes, can form complexes resistant to acid attack in vitro [10,11] as well as in situ [12], through the deposition of a tin-rich surface on the enamel [7]. Zinc fluoride (ZnF_2_), another common ingredient in toothpaste, can provide the antibacterial action of zinc in addition to the cariostatic effect of fluoride [11,13].

Bioactive glasses can be formulated to deliver therapeutic ions that may bond with bone, initiate remineralization, and provide antibacterial effects [14,15,16]. 45S5 Bioglass^®^ was the first inorganic material designed to bond with bone which did not form scar tissue upon implantation in rat femurs [14,17,18]. Bioactive glasses can be doped with ions such as strontium (Sr^2+^) and fluoride (F^−^), which release from the glass to promote remineralization upon degradation [19,20,21]. Antibacterial agents, such as silver and zinc, can also be incorporated into the glass phase [22,23]; their release minimizing further decay. 

Incorporation of bioactive glasses into dentifrices has been reported in the literature, proving the efficacy of these glasses in occluding dentinal tubules to treat or prevent demineralization [24]. NovaMin™, a phosphosilicate-based bioactive glass, has been added to toothpaste, and has been shown to reduce hypersensitivity [25,26,27]. By embedding the glass in a dentifrice such as toothpaste, the dissolution of such glasses results in therapeutic ion release in the environment. This research evaluates the ability of silver-containing glasses, when embedded into a toothpaste, to reduce dental caries. A series of novel silver-containing bioactive glasses were synthesized, which were tailored to be a proactive solution to dental caries. Calcium and phosphorous are incorporated in order to increase remineralization effects, as both are key components of hydroxyapatite (HA); which chemically resembles the mineral component of dentine and enamel [28]. Silver is included in ionic form as it is antibacterial [29,30,31,32,33], even if the mechanism of action is not fully understood [34]. Several attempts to explain the antibacterial effect of silver have been developed [29,34]. Silver has been reported to inactivate metabolism and inhibit bacterial growth [35], uncouple the respiratory chain from phosphorylation [36], interfere with membrane permeability to protons and phosphate [37], and react with thiol groups of membrane-bound enzymes and proteins to inactivate bacteria [34]. It is observed that the most common site of action at low concentrations is the cytoplasmic membrane [38]. At higher concentrations, silver has been observed to interact with cytoplasmic components within the cell [34].

By firing a series of glasses, the effect of different loadings of silver incorporated, and subsequently released in ionic form from a dentifrice, on enamel remineralization and biofilm formation can be determined.

## 2. Results

### 2.1. Network Connectivity

Network connectivity of the three glasses were calculated using Equation (1), and results are shown in Table 1.

(1) $$Network\;Connectivity=\frac{Bridging\;Oxygens-Nonbridging\;oxygens}{Total\;bridging\;species}$$

### 2.2. Glass Characterization

#### 2.2.1. Particle Size Analysis (PSA)

Table 2 contains PSA results. The average particle diameter for all glasses was found to be approximately 4–5 µm. Also, from Table 2, it can be seen that the particle size for all glasses is comparable.

#### 2.2.2. X-ray Diffraction (XRD)

The XRD patterns of the formulated materials provided evidence that all glasses were amorphous. All patterns exhibited a broad hump centered at around 30° 2θ with no crystalline phases present in any of the spectra.

#### 2.2.3. Differential Scanning Calorimetry (DSC)

**Figure 1:**
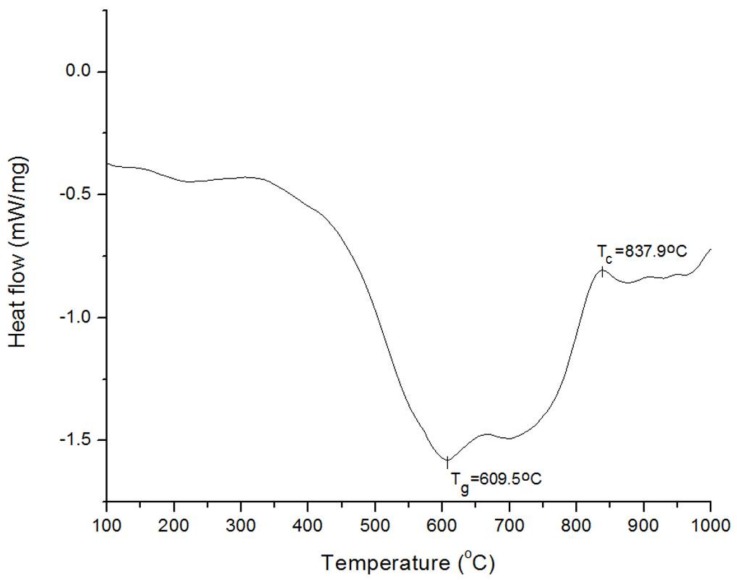
Sample DSC trace for Si-Control glass showing T_g_ and T_c_.

Table 3 summarizes the glass transition (T_g_) and crystallization (T_c_) temperatures of the formulated glasses; a sample DSC trace for the Si-Control glass, is shown in Figure 1.

#### 2.2.4. Scanning Electron Microscopy–Energy Dispersive X-ray Spectroscopy (SEM–EDX)

**Figure 2:**
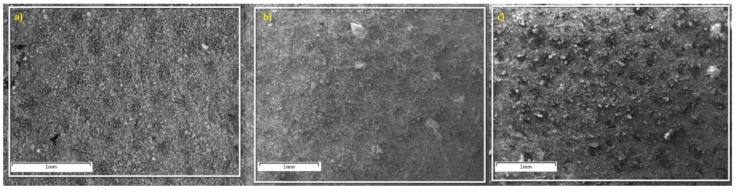
SEM photos of compositions: (**a**) Si-Control (**b**) Si-02 (**c**) Si-05.

SEM images are shown in Figure 2, for (a) Si-Control, (b) Si-02 and (c) Si-05.

EDX traces were taken at multiple sites to determine the composition of the glasses. As can be seen in Table 4, the glass compositions were close to the reagent amounts fired in the crucible.

### 2.3. Solubility Studies

#### 2.3.1. pH Changes

**Figure 3:**
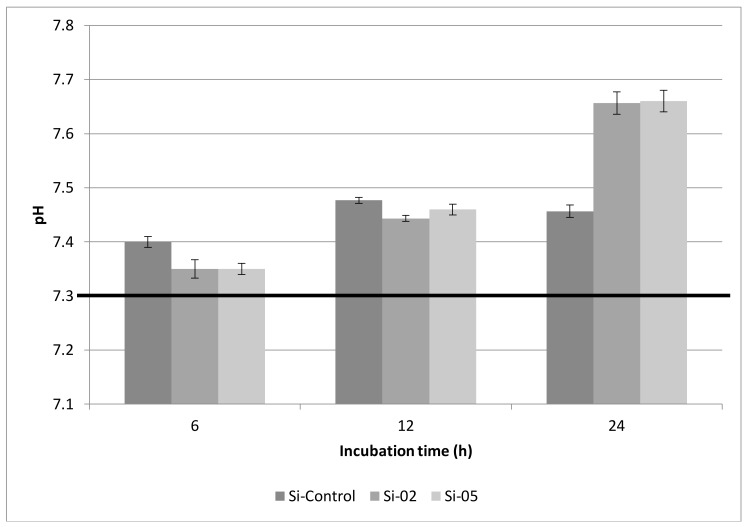
pH response of Tris buffer solution upon glass dissolution after 6, 12, and 24 h. Black horizontal bar represents the initial pH value of the Tris buffer solution before dissolution. Scatter bars indicate standard deviation from the mean.

Figure 3 summarizes the pH results. pH of the Tris buffer solution increased over time as the glass degraded. After 6 and 12 h, the mean pH of the Tris solution was the highest for Si-Control, but it was the lowest after 24 h; Si-02 and Si-05 glasses exhibited statistically-similar pH that was higher than the pH of Si-Control.

#### 2.3.2. Ion Release Studies

**Figure 4:**
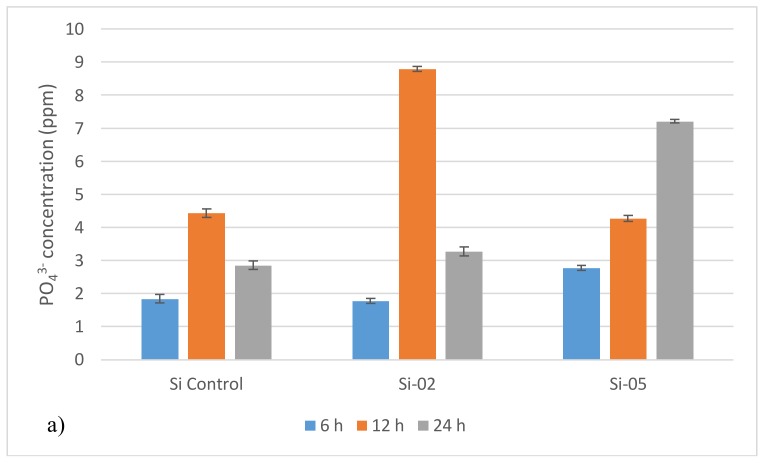

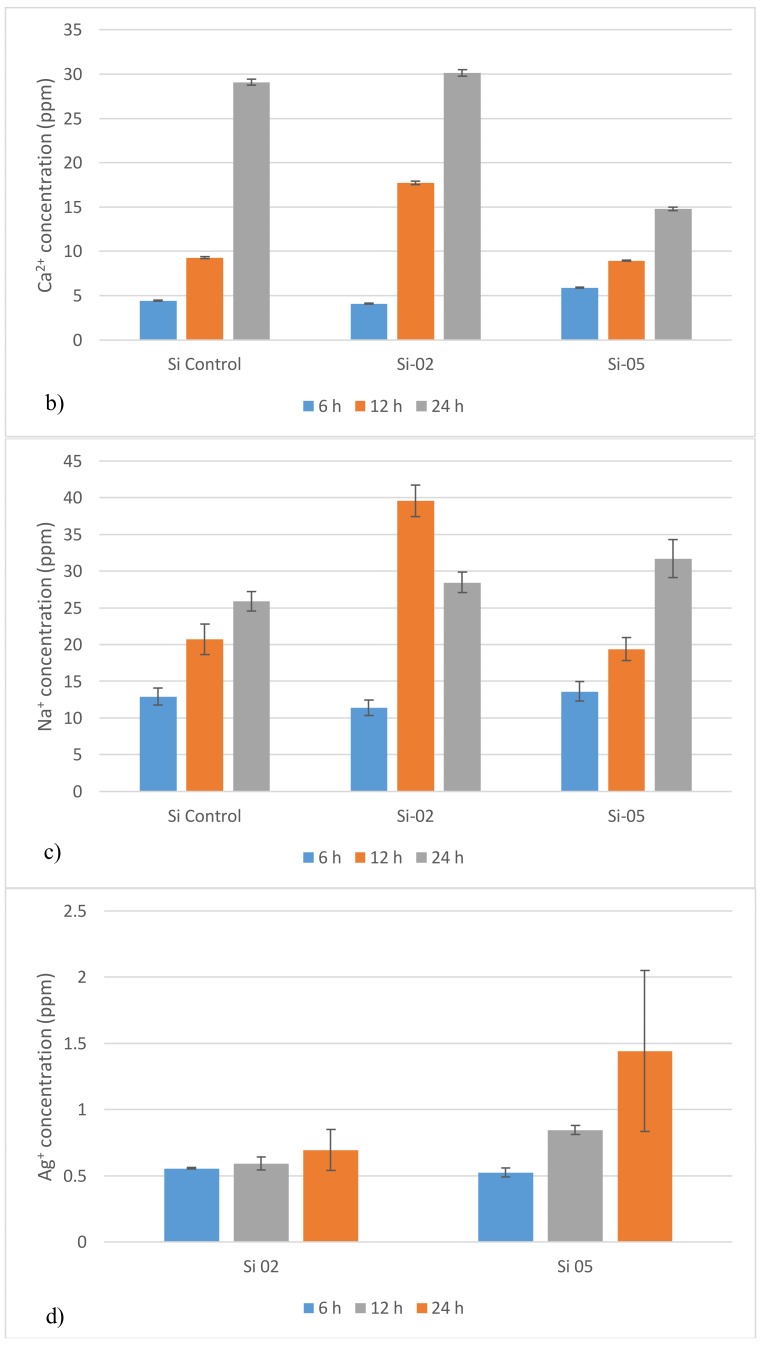
Ion release profiles for (**a**) PO_4_^3−^, (**b**) Ca^2+^, (**c**) Na^+^, and (**d**) Ag^+^. Si-Control Ag^+^ release has been omitted from (**d**) as no silver was present in the composition. Scatter bars indicate standard deviation from the mean.

Figure 4 summarizes the ion release data. Si-05 released more Ag^+^ than Si-02. While all ion amounts increased over time, they did not increase linearly. Si-02, for example, exhibited a spike of PO_4_^3−^ release after 12 h of incubation, with the highest PO_4_^3−^ release compared to Si-Control and Si-05. Si-02 also experienced an increase after 12 h incubation in its Na^+^ release. Ca^2+^ release exhibited more constant ion release rates for all compositions across all time spans.

### 2.4. Antibacterial Studies

**Figure 5:**
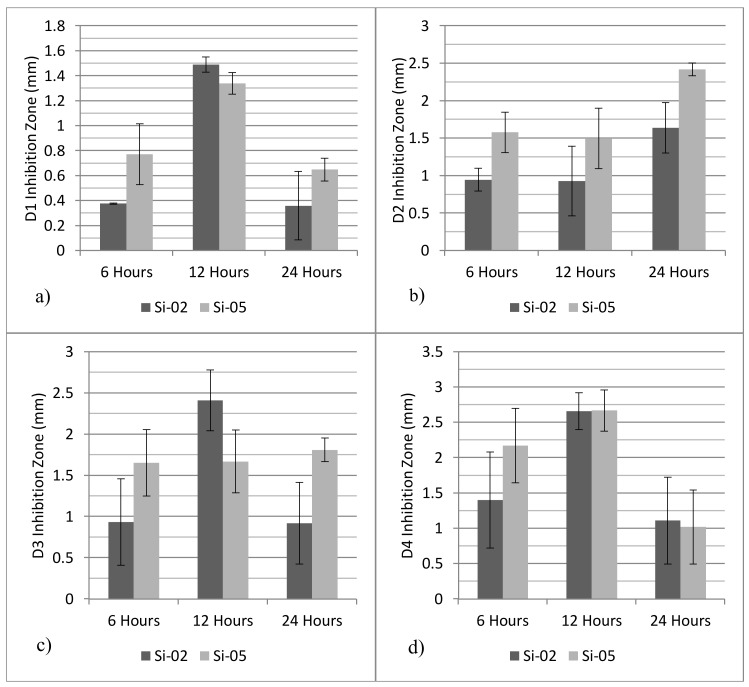
Inhibition zone against bacterial colony D1 (**a**), D2 (**b**), D3 (**c**) and D4 (**d**). The inhibition zone produced by glass powders against four bacterial isolate strains. Si-Control was excluded as it produced no inhibition zone throughout the time period measured. Scatter bars indicate standard deviation from the mean.

Figure 5 presents the inhibition zones against oral bacteria colonies when exposed to the glass powders. As expected, Si-05 exhibited a larger inhibition zone against D2 and D3 strains after 24 h, with statistically similar inhibition zones measured for D1 and D3 strains after 24 h. Additionally, the inhibition zone increased over time for bacteria D2 and D3, whereas D1 and D4 experienced a dip in the inhibition zone from 12 h to 24 h. Furthermore, since the control glasses did not produce any inhibition zone at any time period, the effects of pH rise can be eliminated as a potential bactericidal effect.

### 2.5. Toothpaste Remineralization Studies

**Figure 6:**
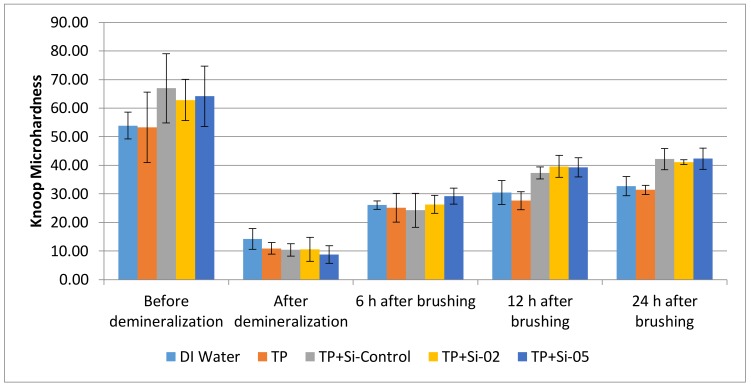
Knoop microhardness remineralization and demineralization on lamb molar dentin samples. All measurements are Knoop hardness (HK). DI is deionized water, TP is toothpaste. Scatter bars indicate standard deviation from the mean.

Figure 6 shows the demineralization and remineralization of dentin molars as a result of their immersion in 1.0 M HCl for 12 h and their subsequent brushing with a glass-embedded toothpaste, respectively. The degree of mineralization was quantified through Knoop microhardness and control measurements were taken after the pucks were prepared and before they were subjected to an acid challenge, as well as before and after their dentifrice remineralization treatments.

**Figure 7:**
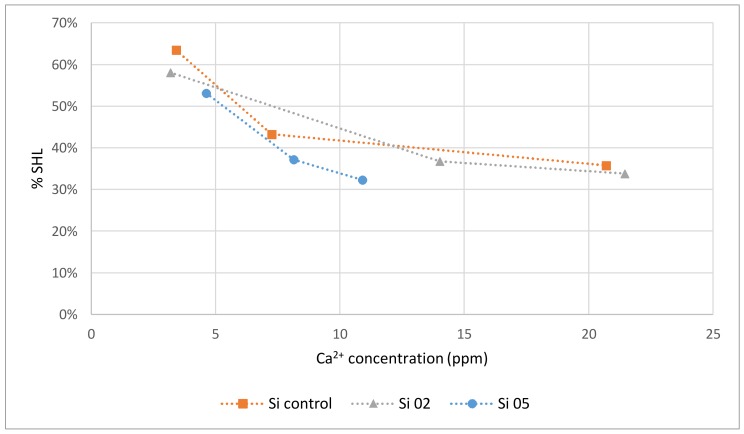
Knoop hardness as a function of Ca^2+^ release from Si-Control, Si-02 and Si-05 glasses.

The percentage of remineralization and demineralization are quantified through percentage hardness loss (%SHL) in Table 5. Percentage hard loss is also plotted against the concentration of Ca^2+^ released per glass, as shown in Figure 7.

## 3. Discussion

Bioactive glasses can be formulated to deliver therapeutic ions that may bond with bone, initiate remineralization, and provide antibacterial effects [14,15,16]. The delivery of ions depends, to a large extent, on the network connectivity of the bioactive material. In the formulated glasses, SiO_2_ is a network former contributing two bridging oxygens, whereas P_2_O_5_, CaO, and Na_2_O are network modifiers. Ag_2_O, however, is a network intermediate. Ca^2+^ provides two non-bridging oxygens, while Na^+^ provides one non-bridging oxygen per Na^+^ ion. Recent work by Hill et al. has shown that P_2_O_5_ functions as an orthophosphate (Q^0^) modifier, contributing three non-bridging oxygen per PO_4_^3−^ ion [39,40]. However, since Ag^+^ ions can function as a network former under certain circumstances, network connectivity has been calculated for the case of Ag^+^ as a network modifier and as a network former. 

Particle sizes were also evaluated. Ideally, the particle sizes are required to be below 20 µm as smaller particle sizes promote faster dissolution due to their greater specific surface area [41]. This allows for the particles to degrade faster, resulting in quicker release of therapeutic ions [42]. The average particle diameter for all glasses was found to be approximately 4–5 µm. This is close to the 3 µm particle size that Mneimne et al. indicate is favorable so that the glass may enter the dentinal tubules, and ultimately, reduce dentine hypersensitivity [43], and as these glasses were designed and proven to be degradable (Section 2.3, Solubility studies), the 4–5 µm diameter would reduce and fit without Mneimne et al.’s criteria. Precipitation of apatite inside the tubules would occlude them and decrease sensitivity. Results show that particle sizes for all glasses are comparable, which means that any changes observed in the glasses’ behavior may be attributed to their different compositions and not to their particle size. SEM images confirmed that all compositions have similar particle sizes. However, morphology, on the small sample size examined, appeared different; Si-05 appears to be more granular and has sharper edges in its particles compared to the more homogeneous scans of Si-Control and Si-02.

DSC results show that T_g_ was not significantly affected by the inclusion of Ag_2_O in the glass network, whereas an increase in T_c_ was first observed at 0.2 mol % Ag_2_O, with a decrease below the T_c_ of the control glass for 0.5 mol % Ag_2_O.

EDX traces confirm that all glass compositions were close to the reagent amounts fired in the crucible. This confirms that the glass thoroughly melted during the firing process, and an increase in silver content is also observed from Si-02 to Si-05.

Based on the pH results, it can be postulated that bioactive glasses can increase the pH of an aqueous solution, such as Tris buffer, upon dissolution. This facilitates apatite formation but can also cause a pH rise too great for the oral environment; an oral pH greater than 7.5 can irritate the oral mucous membranes [43,44]. The Tris buffers containing the Si-02 and Si-05 compositions exhibited a pH of 7.35 at 6 h, while the solution containing Si-Control shows a higher pH rise at 7.4. These values were not determined to be significantly different at *p* < 0.05. After 12 h, the pH responses of the solutions are 7.48, 7.44, and 7.46 for Si-Control, Si-02, and Si-05, respectively, indicating that the advantage gained by the early rise in pH of the Tris solution due to the control glass is lost over longer time frames. Indeed, by 24 h, solutions containing Si-02 and Si-05 both show a pH of 7.66 while the solution containing Si-Control shows a pH of 7.46. This compares favorably with other bioactive glasses in aqueous solution. Brauer et al. found the pH response of a simulated body fluid (SBF) solution containing fluoridated bioactive glass reached 7.6 after 24 h, but plateaued at 7.8 shortly afterward (from day 3 to the end of the experiment at 14 days) [44]. Tris buffer solutions containing Si-02 and Si-05 both reached the same pH after 24 h, 7.66, while the solution containing Si-Control did not show a significant pH rise from 7.48 after 12 h. Time frames greater than 24 h were not examined, as they were not deemed relevant to the application of toothpaste in the oral environment; it is unlikely that the ions deposited from the degrading glass powders would remain in the mouth after a day’s consumption of liquids diluting the contents of the mouth.

These glasses were designed with rapid ion release in mind, and it was observed that, after 12 h, the release rate accelerated. Si-02 releases Ag^+^, on average, 2.5 times faster after 12 h, while Si-05 releases Ag^+^ on average 1.5 times faster after 12 h. The incorporation of silver into the network structure changes the more linear response of the control composition into an accelerating one, although with a slower initial response time. This is due to the complex nature of the network structure when silver is incorporated. As silver oxide is added at the expense of silica, a network modifier is added at the expense of a network former, which should increase bioactivity and solubility of the glass as bridging oxygens are being lost and replaced with non-bridging oxygens facilitating a greater release of cations, specifically sodium and calcium ions, which are basic [45,46,47], and will produce a pH rise. This would disrupt the dissolution mechanism and promote remineralization. However, the initial lag before the 12 h acceleration indicates that the cations are not exchanged as readily during the early stages of dissolution. The complex network may take longer to degrade, and favors dissolution of its formers more than its modifiers. This trend holds up when compared with the ion release profiles obtained in this study. The larger silver content yielded higher initial release volumes, but smaller release rates over time, leading to lower cumulative final release volumes. Interestingly, Si-Control displayed ion release volumes either between the two silver-containing compositions or closer to Si-02 ion profiles. Si-05 releases less silver ions initially, but greater quantities of all other ions when compared with Si-02 after 6 h, with the exception of sodium. However, over time, silver ion release rates increased rapidly for Si-05 while Si-02 released silver ions at a constant rate. Si-02 exhibited higher ion release volumes than Si-05 after 24 h for calcium. It was not possible to measure Silica release, but it may be useful in further explaining how the network degradation functioned as silica was the backbone of the network.

To measure the ion release kinetics of an alkali-silicate glass, the heterogeneous model was used [48,49], which assumes two stages in ionic release: first, the extraction of alkali and silica varies with the square root of time with the exchange of alkali with protons in solution dominating the glass dissolution, then, second, the ion extraction is linear with time. To confirm dissolution behavior under the heterogeneous model, the total silver ion concentration was graphed against the square root of the time, and a linear regression was employed. The correlation coefficient (R2) was above 0.95 for both Si-02 and Si-05 compositions, confirming the model. The network is expected to be complex, formed by a backbone of SiO_4_ tetrahedra connected at the oxygen atoms to form a 3D network. However, the presence of alkali species breaks up the network continuity, hence increasing dissolution, as oxygen atoms no longer bridge to the next tetrahedra, but to an alkali ion instead. As silver ions were added at the expense of silica ions, this would further reduce the network connectivity as network formers were replaced with network modifiers. Ion release rates should be seen to be higher in Si-05 than Si-02, as well as being higher in Si-02 than Si-Control. While this was observed over short time periods, with the exception of silver ion release, longer dissolution time periods favored Si-02.

PO_4_^3−^ and Na^+^ release from Si-02 behaved differently to Ca^2+^ and Ag^+^ release as it reached a peak around 12 h and then diminished after 24 h. The decrease of PO_4_^3−^ and Na^+^ over longer time periods is assumed to be due to a measurement error. This conclusion is based on the fact that phosphorous content is low (3 mol %), and is not expected to release around 9 ppm after 12 h. This will be further investigated in future studies which would focus on pH and ion release of these materials when matured in deionized water, simulated body fluid, and Tris buffer. 

The inhibition zones against oral bacteria show that Si-05 exhibits a larger inhibition zone against D2 and D3 strains after 24 h, with statistically similar inhibition zones measured for D1 and D3 strains after 24 h. Additionally, the inhibition zone increased over time for bacteria D2 and D3, whereas D1 and D4 experienced a dip in the inhibition zone from 12 h to 24 h. Furthermore, since the control glasses did not produce any inhibition zone at any time period, the effects of pH rise can be eliminated as a potential bactericidal effect. Antibacterial studies were singularly affected by Ag^+^ release as all agar discs were stored under similar conditions, and Si-Control exhibited no antibacterial response. It is therefore possible to eliminate pH as a bactericidal factor, since all compositions are known to cause similar pH rises. Referring to bacterial testing results, across all time frames and all bacterial strains, Si-05 outperformed Si-02 in its bactericidal efficacy, which further indicates that silver is the lone bactericidal agent. Silver is known to have wide ranging antibacterial properties; it is believed that DNA stops being able to replicate and proteins become inactive after contact with Ag^+^ [49]; additionally, positively charged silver ions induce an antibacterial effect through electrostatic attraction between negative charged cell membrane of the microorganism and the silver ions [50]. 

Demineralization and remineralization results show that the glass compositions were more effective than the controls, with the highest microhardness after 24 h belonging to toothpaste (TP) + Si-05 followed by TP + Si-Control, TP + Si-02, deionized (DI) water, and finally, TP. However, when taking into account the initial demineralization effects, TP+Si-Control, TP + Si-02 and TP + Si-05 had similar remineralization percentages. Additionally, the response was higher than just the dentifrice treatment alone, indicating that the glass had a positive effect to the mineralization treatments.

Referring to demineralization and remineralization results, all three glasses significantly remineralized enamel after 24 h compared to the demineralized samples. As Si-02 released the greatest amount of calcium and phosphorous after 12 h (17.7 ppm and 8.79 ppm, respectively), samples treated with Si-02 should have exhibited the greatest degree of remineralization at that time frame, which is consistent with the results; from 12 h to 24 h, remineralization of the teeth treated with the toothpaste embedded with Si-02 decreased from 37 to 34% surface hardness loss (%SHL). This study shows that higher Ca^2+^ release resulted in a low %SHL (which is inversely proportional to the % remineralization). There was an upper limit to the amount of remineralization possible after demineralization observed in this experiment after 24 h, which is approximately 34%SHL. To compare, a toothpaste containing 1100 ppm fluoride exhibited a maximum of 38%SHL after four 7-day phases of demineralization/remineralization cycles [51]. Percentage hardness losses for enamel treated with over the counter bleaching agents and then stored in artificial saliva range from 12–40%, according to Zantner et al. [52], while a study by Chen et al. [53] found an 18–32%SHL for enamel treated with fluoridated bleaching agents followed by buffer saline solution. The upper limit can be explained by calcium and phosphorous uptake into the enamel being limited by the concentration of the ions available as the remineralization process requires an excess of calcium and phosphate ions in order to take place [54]: remineralization is hindered by insufficient Ca^2+^ and PO_4_^3−^ in the media. Therefore, there may simply not be enough calcium or phosphorous to repair the lesions and form HA. It is also possible that apatite crystals precipitated over the surface level of the pucks, preventing the penetration of glass deeper into the lesions and remineralization the bulk of the material. However, it is also possible that the reason the teeth did not fully remineralize is due to damage caused by stress propagation to the underlying dentin and cementum during the sample preparation process (grinding and mounting), as well as the resin holding the teeth [55]. Since all compositions had the same amount of calcium and phosphorous, all pucks restored with the glass-loaded dentifrices are expected to reach a similar remineralizing point. While the dentifrice alone was effective at remineralizing the dentine (39 %SHL), the glass loading did increase the remineralization slightly, with a percent difference of 14% between the average glass remineralization and the toothpaste surface hardness loss. However, the difference was not statistically significant at the *p* < 0.05 level.

## 4. Materials and Methods

### 4.1. Glass Synthesis

#### 4.1.1. Glass Fabrication

Three SiO_2_–CaO–P_2_O_5_–Na_2_O_5_–Ag_2_O glasses were synthesized in a series with silver oxide content increased at the expense of silica (Table 6); the quantity of each reagent was selected such that the ratio of calcium to phosphorus (Ca/P) is close to that in the enamel phase of tooth (Ca/P = 1.7) [56] and such that silver oxide is sufficient to be incorporated into the glass network. The glasses were prepared by weighing out appropriate amounts of analytical grade reagents (Fisher Scientific, Ottawa, ON, Canada & Sigma-Aldrich, Oakville, ON, Canada) and firing in a platinum crucible at 1600 °C for 1 h. The melt was subsequently shock-quenched in water, and the resulting glass frit retrieved and dried (24 h, 37 °C). The dried glass was placed in a 50 mL grinding jar and positioned in a Retsch PM100 Planetary Ball Mill (Retsch GmbH, Haan, Germany). Distilled water (25 mL) was added to the grinding jar along with eight 10 mm Ø grinding balls. The remainder of the volume was filled with glass. The jar was placed inside of the mill and allowed to grind at 550 revolutions per minute (RPM) for 15 min. The solution was then removed and placed in a Buchner funnel with a #1 size 9 cm Ø filter paper and a vacuum pressure of about 300–350 mmHg was maintained overnight until the powder was dry. The powder was then scraped off the filter paper and sieved through a 20 µm sieve.

#### 4.1.2. Network Connectivity

Network connectivity (NC) is a measure of the average number of bridging oxygens on each [SiO_4_] tetrahedron [57], which can be used to predict the bioactivity of bioactive glass materials. Generally, the bioactivity increases as NC decreases. NC was calculated using Equation (1). The network formers are assumed to be SiO_2_ and the network modifiers, CaO, Na_2_O, and P_2_O_5_. Ag_2_O generally behaves as a network modifier but can change its role to that of a network former [58]. This usually occurs if Ag_2_O is present in high concentrations, or if metallic silver particles form during glass synthesis. Both cases of silver behaving as a modifier or as a former were considered.

### 4.2. Glass Characterization

#### 4.2.1. Particle Size Analysis (PSA)

A Microtrac S3500 Particle Size Analyzer (Microtrac Inc., Montgomeryville, PA, USA) was used to collect particle size information about the glass powders. Three powder samples per glass were evaluated in the range of 2–60 µm. Results were analyzed by Multisizer software (Version 4, Beckham Coulter, Mississauga, ON, Canada), with means and standard deviations based on counting statistics of 30,000 particles per measurement.

#### 4.2.2. X-ray Diffraction (XRD)

X-ray diffraction (XRD) was performed to confirm that all glass samples were amorphous. Ground samples were placed on a glass slide and inserted into an X’Pert PRO PANanlytical XRD machine (PANanlytic Inc., St Laurent, QC, Canada). Samples were adhered to glass slides and placed inside the machine. Samples were analyzed over the range of 20° ≤ 2θ ≤ 80°, with a step size of 0.05° and a step time of 1.00 s. The generator was set to 40 mA, 45 kV, using a CuKα (1.54 Å) anode.

#### 4.2.3. Differential Scanning Calorimetry (DSC)

Differential scanning calorimetry (DSC) was performed on a NETZSCH STA 449 F3 Jupiter (NETZSCH-Geratebau GmbH, Germany), with an accuracy of 2%, using sapphire crucibles. The analysis was performed for one sample per glass (*n* = 1) from 20 °C to 1000 °C with a step size of 20 °C/min to measure the glass transition (T_g_) and crystallization temperatures (T_c_). For the purpose of this work, T_g_ was chosen as the endset of the inflection in heat flow.

#### 4.2.4. Scanning Electron Microscopy–Energy Dispersive X-ray Spectroscopy (SEM–EDX)

Ground glass samples were placed on a stage using two-sided tape, and excess powder was shaken off. The stage was then placed inside of a JEOL JSM-6380LV scanning electron microscope (JEOL Ltd., Tokyo, Japan). EDX spectra were also collected using a beam energy of 20 kV in order to determine the elements present in the glass. Three different sites were examined and averaged in order to limit the impact of localized testing.

### 4.3. Solubility Studies

A buffer solution was prepared by mixing 15.09 g of Tris (hydroxymethyl) aminomethane in 800 mL of deionized water and adding 44.2 mL of 1 M hydrochloric acid (HCl). The volume was then filled to 2 L using deionized water, and a pH of 7.30 was maintained by adding HCl and testing with a pH meter. The solution was then left at 37 °C overnight. Of each glass powder, 75 mg was added, along with 50 mL of Tris solution in Nalgene plastic bottles. The bottles were then placed in an incubator at 37 °C for 6, 12, and 24 h. The bottles were removed, and the solutions were run through a filter paper to remove undissolved glass. The fluid, meanwhile, was collected and tested for pH and ion release.

#### 4.3.1. pH Change

After 6, 12, and 24 h of incubation of glass powder samples (0.5 g) in Tris buffer solution, the pH of the solution was measured using an Omega PHH222 pH meter (Omega, Laval, QC, Canada) and compared to the initial pH value of the Tris buffer solution to determine how pH varied in the presence of the glass powder.

#### 4.3.2. Ion Release Studies

The collected fluid from the Tris buffer solubility test was evaluated for the presence of silver, calcium, sodium, and phosphate ions (Ag^+^, Ca^2+^, Na^+^, and PO_4_^3−^, respectively) using atomic absorption spectroscopy (AAS, PinAAcle 500 Flame Atomic Absorption Spectrometer (PerkinElmer, Waltham, MA, USA) equipped with a nitrous oxide (N_2_O)/acetylene flame. Calibration standard solutions were prepared for each ion by mixing their corresponding AAS standard (Sigma-Aldrich, Oakville, ON, Canada) in deionized water. AAS standards of 1000 ppm were diluted to 0.10 ppm, 0.50 ppm, 1.0 ppm, 5.0 ppm, 10.0 ppm, and 20.0 ppm. Deionized water was also kept for testing as a blank (0.00 ppm). Each sample was tested three times. 

### 4.4. Antibacterial Studies

Oral cavity bacteria were collected by swabbing the teeth, gums, and tongues of three volunteers (all male, healthy, and ages 25, 25, and 24 years old) at Ryerson University (Toronto, ON, Canada). The swabs were then placed in nutrient broth and allowed to grow for 3 days, after which they were inoculated onto strep agar plates, and left to grow for a further 3 days. Four colonies of interest, such as those exhibiting satellitism or microcolonies, were then extracted and suspended in broth again for another three days; these colonies are expected to be formed of *Staphylococcus*, *Streptococcus*, and *Lactobacillus* genus bacteria, typically found in healthy mouths [59]. These isolates were then streaked across agar plates with glass powder. These agar plates were divided into three sections, and 0.5 g of each glass composition measured out in Eppendorf tubes were placed in each third. After 6, 12, and 24 h, the inhibition zone was measured for each composition by comparing the dark, inoculated spaces against the lighter, inhibited spaces, and measuring using a caliper. The difference in the diameters of the inhibited zone and the glass deposit was measured.

### 4.5. Toothpaste Remineralization Studies

**Figure 8:**
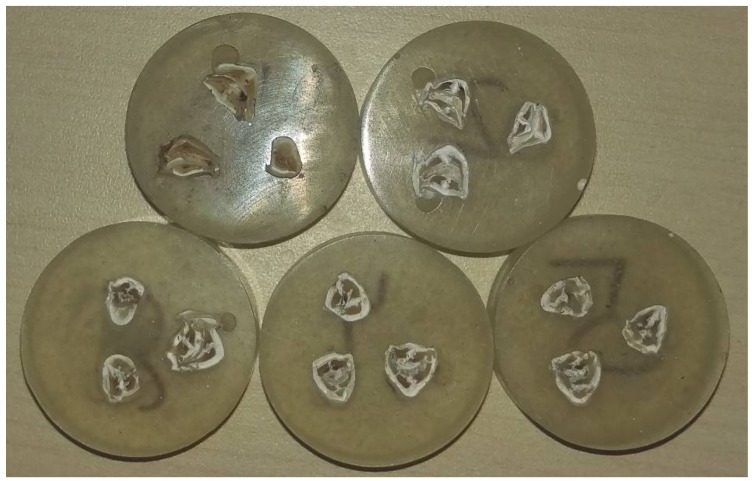
Dental pucks mounted in resin and with their buccal surfaces exposed. Picture was taken after they were brushed.

Fifteen lamb molars were obtained from a local butcher, and their buccal surfaces ground with 600 grit sandpaper on a water-cooled disc grinder. The molars were then mounted, three per puck, in cold-curing resin, with the buccal surfaces facing upward. The resin was then ground down with 400, 600, and 1200 grit sandpaper, successively, on a water-cooled bench disc grinder (Buehler Ltd., Lake Bluff, IL, USA). The microhardness of the buccal surfaces were determined using an HM-114 Mitutoyo Testing Machine (Mitutoyo, Mississauga, ON, Canada) equipped with a Knoop indenter. The test was performed by loading the samples with a force of 1 kgf (9.81 N) for 10 s and measuring the size of the indentation using SEM images. All five pucks were then submerged in 1.0 M HCl for 12 h at 37 °C to simulate demineralization. The pucks were then retested for their microhardness, and SEM images of the buccal surfaces were captured. Subsequently, three of the pucks were randomly selected for remineralization through brushing with a glass-embedded toothpaste (1g toothpaste with 0.5 g of glass) while two pucks were selected for control. One control involved brushing employing a glass-free dentifrice (1 g), while the other involved brushing with deionized water. All pucks were brushed for 1 min and only once. After remineralization, microhardness testing was performed again at 6, 12, and 24 h, and SEM images were recorded using a JSM-6380LV SEM (JEOL Ltd., Tokyo, Japan). The pucks are shown in Figure 8.

The degree of remineralization was quantified by the percentage of surface hardness loss (%SHL), a standard metric commonly used to describe mineral loss during dissolution as a function of microhardness [60]. A lower %SHL is desirable, as it indicates less mineral loss. %SHL can be calculated using Equation (2).

(2) $$\%SHL=\frac{Baseline\;Hardness-Treated\;Hardness}{Baseline\;Hardness}\times 100\%$$

### 4.6. Statistical Analysis

Non-parametric Kruskal–Wallis H Test was used to analyze the data. The Mann–Whitney U test was used to compare the relative means and to report the statistically significant differences when *p* ≤ 0.05. Statistical analysis was performed on all groups where *n* ≥ 3. Statistical analysis was performed using SPSS software (IBM SPSS statistics 21, IBM Corp., Armonk, NY, USA). 

## 5. Conclusions

Three glass compositions were synthesized and evaluated to determine effectiveness as anti-caries components when incorporated into dentifrices. The glasses were characterized and tested for their antibacterial properties as well as their enamel remineralization capability.

- The addition of 0.2 and 0.5 mol % silver oxide to a SiO_2_–CaO–P_2_O_5_–Na_2_O glass composition at the expense of silica did not cause the glasses to crystallize when fired through the melt-quench method.
- The glasses caused a pH response in Tris buffer solution, raising pH from 7.3 to approximately 7.6. This is invaluable as remineralization and demineralization are pH dependent processes. An acidic environment will promote demineralization, while a more alkaline environment will be more conducive to remineralization. However, an excessive increase in oral pH, typically over 7.5, can cause irritation.
- Ion release profiles were found to be dependent on time and silver content. The network structure of the glass is complex and glass dissolution follows the heterogeneous model, meaning the release of silver occurs in two phases; the first involving alkali ions being replaced with hydronium ions, which varies with the square root of time, while the second is the dissolution of the network, which varies linearly with time.
- Si-05 glass exhibited a stronger antibacterial effect than the Si-02 glass, while Si-Control exhibited no antibacterial effect. This stronger antibacterial response was due to the silver content in each composition; while pH changes are caused by all glasses, Si-Control contains no silver. It can be said then that Ag^+^ is the sole bactericidal factor in the glasses.
- All glasses remineralized lamb dental enamel when incorporated into a dentifrice that the molars were exposed to post a standard in vitro demineralization process. This remineralization effect was more potent than the toothpaste alone, which itself is more effective than deionized water. All compositions contain the same amount of calcium and phosphorous. As these are the constituent elements in HA, it is believed that Ca^2+^ and PO_4_^3−^ cause the remineralization effect. This section is not mandatory, but can be added to the manuscript if the discussion is unusually long or complex.

**Limitations:**

- The proposed formulations for the glasses involved higher mole percentages of silver oxide than those synthesized; however, it was determined, upon firing such glasses, that in order for the glass to fully incorporate the silver oxide in its structure, the amount of silver oxide had to be reduced to a maximum of 0.5 mol % Ag_2_O.
- The amount of silicon ions released after incubating the glass samples was not measured; in future works, silicon ion release is necessary, as this may be useful to explain how the glass network degradation functioned as silica was the backbone the here-in proposed silver-containing glasses.
- Incubation studies in SBF should be performed to evaluate the deposition of calcium phosphate (CaP) on the teeth surface.
- The ion release studies performed in this study show some unexpected results particularly for PO_4_^3−^ and Na^+^. The non-linear results obtained for Si-02 samples could be due to machine error. Another study will be performed to provide further insight into the ion release of the formulated materials when matured in de-ionized water, SBF and Tris buffer solutions.

## Figures and Tables

**Table 1:** Network connectivity for each composition (mol %) assuming silver is either a network modifier (NM) or network former (NF).

Glass	NM	NF
Si-Control	1.314	1.314
Si-02	1.309	1.315
Si-05	1.302	1.317

**Table 2:** Particle size analysis (PSA) results. All measurements are in micrometers (µm). d_10_, d_50_, and d_90_ indicate the 10%, 50%, and 90% diameter on the cumulative volume distribution, respectively.

Glass	d_10_	d_50_	d_90_
Si-Control	2.29	4.22	9.31
Si-02	2.29	4.31	10.33
Si-05	2.38	5.11	12.73

**Table 3:** Glass transition and crystallization temperatures of glass compositions. Variation was based on equipment accuracy of 2%.

Glass	T_g_ (°C)	T_c_ (°C)
Si-Control	610 ± 12	838 ± 17
Si-02	618 ± 12	848 ± 17
Si-05	620 ± 12	826 ± 17

**Table 4:** Average of wt % in compositions for each composition.

Element	Si-Control	Si-02	Si-05
O	50.6	54.8	54.7
Si	29.0	25.9	24.9
Na	10.3	10.3	10.6
Ca	7.60	6.60	6.77
P	2.50	2.03	2.03
Ag	0	0.3	0.97

**Table 5:** Percent surface hardness loss (%SHL) for each stage of treatment as compared with baseline Knoop values.

Treatment	After Demineralization	6 h after Brushing	12 h after Brushing	24 h after Brushing
DI Water	73%	51%	43%	39%
TP	79%	52%	47%	39%
TP + Si-Control	84%	64%	43%	36%
TP + Si-02	83%	58%	37%	34%
TP + Si-05	86%	53%	37%	32%

**Table 6:** Silica glass series formulations (mol %).

Glass	SiO_2_	CaO	P_2_O_5_	Na_2_O	Ag_2_O
Si-Control	70	12	3	15	0
Si-02	69.8	12	3	15	0.2
Si-05	69.5	12	3	15	0.5
